# Assessing the reliability of AI-generated responses to patient-oriented lung cancer questions: a multi-platform study

**DOI:** 10.1186/s12890-026-04903-x

**Published:** 2026-10-23

**Authors:** Erhan Özer, Gizem Gedikoğlu Pirim

**Affiliations:** 1 Department of Thoracic Surgery, Bursa Uludağ University Faculty of Medicine, 1st Floor, 16059 Bursa, Turkey; 2 Department of Thoracic Surgery, Afyon State Hospital, Afyon, Turkey

**Keywords:** Large language models, Artificial intelligence, Lung cancer, Patient education, Readability, Reproducibility, Clinical relevance, Health literacy

## Abstract

**Background:**

Large language models (LLMs) are increasingly used by patients seeking lung cancer–related medical information. Recent studies have increasingly compared multiple LLMs for patient-facing medical information; however, differences in clinically relevant performance dimensions and consistency across repeated responses remain important areas for evaluation.

**Methods:**

Seventy-nine lung cancer–related patient questions were queried across three freely accessible LLM platforms (ChatGPT, Claude, and Google Gemini) between December 2025 and January 2026. Responses were independently evaluated by two specialist thoracic surgeons for accuracy, completeness, clinical relevance, and readability, with disagreements resolved by consensus. Reproducibility was assessed through five repeated independent queries using a clinical-equivalence framework, and automated readability was assessed using the Flesch–Kincaid Grade Level. Ordinal outcomes were analyzed using cumulative link mixed models accounting for repeated evaluation of questions, while reproducibility was analyzed using generalized estimating equations.

**Results:**

All platforms demonstrated high accuracy, with no significant overall platform difference (*p* = 0.150). Completeness differed significantly across platforms (*p* < 0.001), with higher ratings for ChatGPT than Claude and Gemini and for Claude than Gemini. Clinical relevance showed no overall platform effect (*p* = 0.568), although a platform-by-domain interaction was observed (*p* = 0.046). Expert-rated readability differed significantly (*p* < 0.001), with Gemini receiving higher ratings than ChatGPT and Claude. All platforms produced content requiring approximately 10th–12th grade reading levels. Reproducibility was high overall (88.6–96.2%), with no statistically significant overall platform effect (*p* = 0.072).

**Conclusions:**

Contemporary LLMs demonstrated high accuracy and clinical relevance, but differed in completeness and readability. Reproducibility was high across platforms, while all models exceeded recommended patient reading levels. Further patient-based validation is needed before routine clinical integration.

**Clinical trial number:**

Not applicable

**Supplementary Information:**

The online version contains supplementary material available at https://doi.org/10.1186/s12890-026-04903-x.

## Introduction

Lung cancer remains a leading cause of cancer-related mortality worldwide [1, 2]. Despite advances in early detection and treatment, nearly 57% of patients still present with advanced-stage disease (III–IV), and 5-year survival for stage IV remains below 10% [3]. As a result, patients and caregivers increasingly seek information regarding diagnosis, treatment options, and prognosis.

Patterns of medical information seeking have changed substantially. Beyond physician–patient interactions, internet-based resources have become a major source of health information. More recently, artificial intelligence–driven conversational platforms based on large language models (LLMs) have emerged as easily accessible tools that generate rapid, seemingly personalized medical information, thereby influencing patient expectations and treatment-related decisions [4, 5].

Recent research has increasingly moved from single-model assessments toward comparative evaluations of multiple contemporary LLMs. In oncology, multi-model studies have examined patient-oriented information across dimensions including factual accuracy, completeness, readability, reliability, understandability, actionability, and expert-rated quality. Recent lung cancer–specific evaluations have likewise compared several publicly available LLMs using standardized questions or clinical scenarios, demonstrating model-dependent differences in accuracy, readability, and patient-centered information quality. These findings indicate that cross-platform evaluation is now an established component of LLM assessment rather than an unexplored area. Nevertheless, performance across different quality dimensions is not necessarily concordant, and consistency across repeated generations remains an additional consideration when evaluating patient-oriented medical information [6–9] .

Against this evolving evidence base, we performed a multi-platform, multidimensional evaluation of AI-generated responses to 79 patient-oriented lung cancer questions across ChatGPT 5.2, Claude Sonnet 4.5, and Google Gemini 3. Responses were assessed by two thoracic surgeons across accuracy, completeness, clinical relevance, readability, and reproducibility. In addition to comparing these quality dimensions using a common question set, we evaluated clinical consistency across five independent responses to each question on each platform.

## Materials and methods

### Ethical considerations and disclosures

This study involved the analysis of AI-generated textual responses to standardized, publicly available patient-oriented questions and did not include human participants, patient records, or identifiable personal data. Accordingly, formal institutional review board or ethics committee approval was not applicable. The study was conducted in accordance with general principles of research integrity and responsible AI evaluation.

All analyzed data consisted exclusively of AI-generated responses to predefined prompts. The authors declare no financial relationships, conflicts of interest, or institutional affiliations with OpenAI, Anthropic, or Google. AI-assisted tools (ChatGPT and Grammarly) were used solely for language editing and grammatical refinement of the manuscript. No AI tools were used for data generation, data analysis, interpretation of results, or scientific decision-making.

### Question development and selection

The question set was developed in December 2025 using a structured, multistep process designed to capture common patient-oriented information needs across the lung cancer care continuum. Candidate questions were first identified through Google Search using the query “questions about lung cancer” and through related questions displayed in the “People also ask” section. To broaden the scope beyond search-engine–derived questions and ensure clinically relevant topic coverage, patient-oriented resources from the American Cancer Society, National Cancer Institute, and MedlinePlus Medical Encyclopedia were also reviewed. These sources were selected to capture commonly encountered information needs among individuals seeking lung cancer information online. Search-engine queries were used as a proxy for frequently sought public information, while established patient-oriented resources were included to ensure clinically relevant coverage across the lung cancer care continuum. The size of the question corpus was determined by the structured question-selection process and breadth of topic coverage rather than by a statistical power calculation. No formal a priori sample-size or power calculation was performed for the between-platform comparisons.

All candidate questions were independently screened by two investigators. Questions were excluded if they were (1) duplicates or semantically overlapping with another question (2), subjective or dependent on individual patient characteristics (3), vague or clinically non-specific, or (4) related primarily to institutional or physician preference rather than general lung cancer information. Disagreements regarding eligibility were resolved by consensus. Following screening and removal of overlapping or ineligible questions, 79 unique patient-oriented questions were retained for analysis.

Before submission to the LLMs, the final question set was independently categorized according to its primary clinical content into four predefined domains: Basic Knowledge (*n* = 18), Diagnosis and Staging (*n* = 16), Treatment Options (*n* = 22), and Prognosis, Risks, and Follow-up (*n* = 23). Categorization disagreements were resolved by consensus. Representative questions from each domain are presented in Table 1, and the complete 79-question dataset, organized by domain, is provided in Supplementary Table S1 to ensure transparency and reproducibility of the question-selection framework.

### Large language model selection and querying protocol

Three large language models were evaluated using freely available public interfaces between December 15, 2025 and January 1, 2026: ChatGPT 5.2 (OpenAI), Claude Sonnet 4.5 (Anthropic), and Google Gemini 3 (Alphabet Inc.). All platforms were accessed through free-access accounts via a web browser from Türkiye. The model versions were recorded exactly as displayed to end users by the respective platforms at the time of data collection and therefore represent provider-declared, user-facing model names rather than API model identifiers.

All queries were performed through the standard user-facing web interfaces rather than APIs. Questions were entered in English as plain text without additional prompting, role assignment, contextual information, or prompt engineering. Web browsing/search functions were not intentionally activated or requested, and no external sources were supplied to the models. Default platform settings were retained, with no manual modification of available safety settings or provider-level safeguards. No custom instructions or other user-defined personalization settings were applied. Because the models were accessed through proprietary user-facing web interfaces, generation parameters that may influence stochasticity, including temperature, top-p, and random seed, were not exposed to users and therefore could not be standardized or recorded.

To minimize contextual carryover and maximize independence between responses, each question was submitted in a new browser session with cache and cookies cleared. The same standardized querying procedure was applied to all 79 questions across all three platforms. All platform-generated responses were retained and analyzed in their original, unedited form, with no content removed before evaluation. Accordingly, any platform-generated disclaimers, warnings, or safety statements were retained and included in word-count and readability calculations. Because the questions were derived from publicly accessible search results and patient-oriented health resources, prior exposure of identical or conceptually similar content during model training could not be determined, as the complete training corpora of the proprietary models are not publicly available.

Reporting of the LLM evaluation protocol was reviewed against contemporary LLM-specific reporting recommendations, including CHART and MI-CLEAR-LLM, with applicable items incorporated into the description of model identification, access mode, prompt execution, platform settings, repeated querying, and response evaluation [10, 11].

### Evaluation framework for AI-generated responses

AI-generated responses were independently evaluated by two thoracic surgeons with expertise in lung cancer management across five quality dimensions: accuracy, completeness, clinical relevance, readability, and reproducibility. The evaluation framework was adapted from validated methodologies used in prior studies assessing AI-generated medical content [12–14].

#### Reviewer training and evaluation process

Prior to formal evaluation, both reviewers independently assessed pilot responses to calibrate scoring criteria and ensure consistency. Responses were presented in randomized order with platform identifiers masked; however, complete blinding was not feasible due to distinctive formatting characteristics. To minimize recognition bias, reviewers were instructed to focus exclusively on factual content and clinical applicability.

Accuracy, completeness, clinical relevance, and readability were independently scored by both reviewers. Disagreements, defined as ratings differing by more than one point, were resolved through consensus discussion. Inter-rater agreement between the two reviewers for the four-point ordinal ratings of accuracy, completeness, clinical relevance, and readability was assessed using Cohen’s kappa with quadratic weights, which assigns progressively greater penalties to larger disagreements between ordinal categories. Kappa values were interpreted as < 0.00, poor; 0.00–0.20, slight; 0.21–0.40, fair; 0.41–0.60, moderate; 0.61–0.80, substantial; and 0.81–1.00, almost perfect agreement. Kappa estimates are reported with bootstrap 95% confidence intervals based on 2,000 resamples.

Reproducibility (i.e., consistency across repeated responses to the same question) was evaluated jointly by both reviewers based on clinical equivalence across all five response iterations. Reproducibility in this analysis therefore represents model response consistency across repeated generations and should not be interpreted as a measure of reviewer-assessment reliability. Because clinical equivalence was determined jointly by the two reviewers as a binary outcome, inter-rater reliability for the reproducibility classification was not independently estimated.

#### Quality metrics: definitions and scoring

A total of 1,185 responses were generated (79 questions × 3 AI platforms × 5 independent queries). For the assessment of accuracy, completeness, clinical relevance, and readability, only the first response generated for each question on each platform was evaluated, resulting in 237 primary responses (79 questions × 3 platforms). This analytic approach was specified before formal scoring. The first response was designated as the standardized primary response for cross-platform comparison of quality metrics, whereas the five repeated responses were generated and retained specifically to assess within-platform response consistency. Accordingly, repeated responses were not treated as additional observations for the primary quality assessments. All five responses per question and platform were retained and used for the reproducibility assessment, as described below.

Accuracy, completeness, and clinical relevance were treated as related but conceptually distinct dimensions. Accuracy assessed whether the information provided was factually and clinically correct based on contemporary evidence-based clinical knowledge, including current authoritative textbooks, peer-reviewed literature, and established clinical practice standards. The two expert reviewers independently evaluated each response using these sources and their specialist clinical expertise. Thus, a response could be accurate but incomplete if the information presented was correct but important elements were omitted.

Completeness assessed the extent to which the response covered the key informational components required to adequately answer the question, irrespective of whether all included information was clinically useful to an individual patient. For example, a response could contain extensive and accurate information but still receive a lower completeness score if an essential aspect of the question was not addressed. To improve transparency regarding the core clinical content considered relevant to each question, the reviewers subsequently compiled question-specific key points for accuracy and completeness after completion of the independent assessments. These key points are provided for all 79 questions in Supplementary Table S1.

Clinical relevance assessed whether the information provided was directly applicable and useful for patient understanding or decision-making in a typical lung cancer care context. Accordingly, a response could be accurate and comprehensive yet receive a lower clinical relevance score if it contained substantial information that was technically correct but peripheral to the patient’s question or unlikely to influence patient understanding or clinical expectations.

These distinctions were maintained throughout the evaluation: accuracy addressed correctness (“Is the information correct?”), completeness addressed coverage (“Are the key elements addressed?”), and clinical relevance addressed applicability (“Is the information useful and pertinent to the patient’s question?”). Each dimension was scored independently using the predefined 4-point criteria, as detailed in Table 1.

Readability was defined as the linguistic complexity and ease of reading of the generated text and was considered distinct from understandability, comprehensibility, and actionability. The Flesch–Kincaid Grade Level was used as an objective measure of linguistic complexity and was not interpreted as a direct measure of patient comprehension or actionability.

#### Reproducibility assessment protocol

Reproducibility (i.e., consistency across repeated responses to the same question) was defined as the ability of an AI model to provide consistent clinically equivalent responses to identical questions across repeated, independent queries. Consistency was assessed using clinical equivalence rather than textual similarity, reflecting the practical requirement that repeated AI-generated responses should lead to the same patient understanding or clinical expectation.

For reproducibility assessment, responses were considered clinically equivalent when they conveyed the same core recommendations or key informational content such that a patient would be expected to reach the same understanding or clinical expectation, despite minor differences in wording or structure. Minor variations in wording, structure, or emphasis without clinical impact were considered clinically equivalent and did not affect reproducibility classification.

All 79 questions were queried five times per AI platform to assess response variability, and all generated responses were retained. Repeat queries were performed in new browser sessions with cleared cache and cookies to ensure independence from contextual memory effects. For the final reproducibility assessment, all five stored responses for each question were evaluated jointly by the two expert reviewers for clinical equivalence, and no early-stopping procedure was applied. The responses were reviewed sequentially from the first through the fifth response. If any subsequent response showed a clinically meaningful deviation from the preceding responses that could alter patient understanding or clinical expectations, the question was classified as non-reproducible; however, the remaining responses were still evaluated. Reproducibility was assessed as a binary outcome. A question was classified as reproducible only if all five responses remained clinically equivalent in their core information and recommendations. This conservative binary approach was selected from a patient-safety perspective, based on the premise that even a single clinically meaningful deviation across repeated responses may be relevant when patients re-query the same information.

Automated semantic similarity metrics were not incorporated because lexical or embedding-based similarity does not necessarily correspond to clinical equivalence: responses with different wording may convey clinically equivalent information, whereas relatively subtle semantic differences may have clinically meaningful implications. Expert assessment was therefore prioritized for identifying clinically relevant inconsistency.

**Table 1:** Scoring criteria for quality assessment of AI-generated responses to lung cancer patient questions

Grading Category	Definition
Accuracy (1–4 scale)	1 = Completely incorrect or potentially harmful information. 2 = Partially correct with major omissions or errors affecting interpretation 3 = Mostly correct with minor inaccuracies not affecting interpretation 4 = Fully accurate and clinically appropriate.
Completeness (1–4 scale)	1 = Missing essential information; inadequate for patient understanding. 2 = Basic information provided but lacking important details. 3 = Comprehensive coverage with minor gaps. 4 = Complete and thorough, addressing all key aspects.
Clinical Relevance (1–4 scale)	1 = Not clinically relevant or applicable. 2 = Limited clinical relevance. 3 = Relevant for most patients in typical scenarios. 4 = Highly relevant and directly applicable to patient care.
Readability (1–4 scale)	1 = Very difficult (college level or above). 2 = Difficult (high school level). 3 = Moderately difficult (8th–9th grade level). 4 = Easy to understand (6th grade level or below).
Reproducibility (Binary)	**Reproducible**: Clinically equivalent responses across all iterations, with no meaningful impact on clinical recommendations. **Non-reproducible**: Clinically meaningful variation detected at any iteration, potentially affecting clinical decisions.

Scoring criteria used for the quality assessment of AI-generated responses to lung cancer–related patient questions. Responses were evaluated across four domains (accuracy, completeness, clinical relevance, and readability) using a 4-point Likert scale, while reproducibility was assessed as a binary outcome.

### Objective performance metrics

In addition to qualitative evaluation, two objective performance metrics were recorded for each AI-generated response. Response time was measured in seconds from query submission until the platform indicated completion of response generation. Word count was obtained using Microsoft Word’s built-in word count tool. These metrics provided complementary insight into response efficiency and verbosity.

### Statistical analysis

Statistical analyses were performed using IBM SPSS Statistics version 28.0 (IBM Corp., Armonk, NY, USA) and R version 4.5.3 (R Foundation for Statistical Computing, Vienna, Austria). Continuous variables are presented as mean ± standard deviation or median with interquartile range, as appropriate, and categorical variables as frequencies and percentages. Inter-rater agreement between the two reviewers for the four-point ordinal ratings of accuracy, completeness, clinical relevance, and readability was assessed using quadratic-weighted Cohen’s kappa.

The primary inferential analyses accounted for the repeated evaluation of the same questions across AI platforms. Accuracy, completeness, clinical relevance, and readability were analyzed using cumulative link mixed models with a logit link. The original 1–4 ratings from both reviewers were retained as ordinal outcomes rather than averaged scores. Platform and clinical domain were included as fixed effects, reviewer was included as a fixed effect, and question was modeled as a random intercept to account for question-level heterogeneity and repeated observations across platforms. Models were fitted using adaptive Gauss–Hermite quadrature. The overall contribution of platform was evaluated using likelihood-ratio tests comparing models with and without the platform term.

Platform-by-domain interactions were evaluated by comparing additive and interaction models using likelihood-ratio tests. When no significant interaction was identified, the additive model was retained for interpretation. Pairwise platform contrasts were estimated from the fitted models, and multiplicity was controlled using the Holm method. For significant platform-by-domain interactions, domain-specific platform contrasts were examined, with Holm correction applied across the complete set of domain-specific comparisons. Effect estimates are reported as proportional odds ratios (ORs) with 95% confidence intervals.

Reproducibility was analyzed as a binary outcome at the question-platform level, with each observation representing clinical consistency across all five repeated responses. Platform-specific reproducibility proportions are reported with Wilson 95% confidence intervals. A mixed-effects logistic regression with question as a random intercept was initially considered; however, the random-effect variance was estimated as zero, resulting in a singular fit. Because sparse non-reproducible events and complete separation within the prognosis domain further limited mixed-model estimation, reproducibility was analyzed using logistic generalized estimating equations with question as the clustering unit and an exchangeable working correlation structure. Platform and domain were included as predictors, and platform effects are reported as odds ratios (ORs) with 95% confidence intervals. Platform-by-domain interaction estimates were not interpreted because separation resulted in unstable parameter estimates.

Automated readability assessed using the Flesch–Kincaid Grade Level, response time, and word count were compared across platforms using the Friedman test. When appropriate, post hoc pairwise comparisons were performed using Wilcoxon signed-rank tests with Bonferroni correction. Correlations between response characteristics and quality metrics, as well as associations between expert readability ratings and Flesch–Kincaid Grade Levels, were assessed using Spearman’s rank correlation coefficient. These analyses were considered secondary or exploratory. All tests were two-sided, and a *p* value < 0.05 was considered statistically significant.

## Results

A total of 1,185 responses were generated across the three AI platforms (79 questions × 3 platforms × 5 independent queries). Of these, 237 primary responses, representing the first response to each question on each platform, were evaluated for accuracy, completeness, clinical relevance, and readability. All 1,185 responses were included in the reproducibility assessment. Question distribution by category was: Basic Knowledge (*n* = 18, 22.8%), Diagnosis and Staging (*n* = 16, 20.3%), Treatment Options (*n* = 22, 27.8%), and Prognosis, Risks, and Follow-up (*n* = 23, 29.1%).

Inter-rater agreement between the two reviewers was assessed using quadratic-weighted Cohen’s kappa. Substantial agreement was observed for accuracy (κ = 0.782, 95% CI 0.721–0.836), completeness (κ = 0.651, 95% CI 0.573–0.716), and clinical relevance (κ = 0.787, 95% CI 0.713–0.852), while agreement for readability was almost perfect (κ = 0.963, 95% CI 0.945–0.977) (all *p* < 0.001).

### Accuracy, completeness, and clinical relevance

Overall accuracy and clinical relevance scores were high across all three platforms, whereas greater between-platform variation was observed for completeness (Table 2a). In the cumulative link mixed models, there was no significant overall platform effect on accuracy (likelihood-ratio χ²(2) = 3.79, *p* = 0.150), and no platform-by-domain interaction was observed (χ² (6) = 5.91, *p* = 0.434). Completeness differed significantly across platforms (likelihood-ratio χ² (2) = 55.36, *p* < 0.001), without a significant platform-by-domain interaction (χ² (6) = 11.43, *p* = 0.076). Clinical relevance showed no significant overall platform effect (likelihood-ratio χ² (2) = 1.13, *p* = 0.568), although a significant platform-by-domain interaction was observed (χ² (6) = 12.84, *p* = 0.046) (Table 3b). No responses were rated as completely incorrect on the accuracy scale.

Post hoc comparisons based on the cumulative link mixed models showed that completeness was significantly higher for ChatGPT than Claude (OR 1.98, 95% CI 1.17–3.35; Holm-adjusted *p* = 0.011) and Gemini (OR 7.01, 95% CI 4.02–12.23; *p* < 0.001), while Claude also had higher completeness than Gemini (OR 3.54, 95% CI 2.10–5.97; *p* < 0.001). For clinical relevance, the significant platform-by-domain interaction was primarily attributable to the prognosis domain, in which ChatGPT had higher odds of receiving a higher rating than Gemini (OR 5.05, 95% CI 1.89–13.47; Holm-adjusted *p* = 0.015); no other domain-specific comparison remained significant after multiplicity adjustment (Table 3b).

**Table 2:** a. Descriptive scores across platforms and domains. B. Model-based comparisons

Domain	Metric	ChatGPT	Claude	Gemini
**Overall**	**Accuracy**	3.56 ± 0.62; 4 (3–4)	3.49 ± 0.68; 4 (3–4)	3.59 ± 0.57; 4 (3–4)
	**Completeness**	3.38 ± 0.65; 3.5 (3–4)	3.25 ± 0.64; 3 (3–4)	2.99 ± 0.61; 3 (2.5–3.5)
	**Clinical relevance**	3.46 ± 0.68; 4 (3–4)	3.42 ± 0.68; 3.5 (3–4)	3.41 ± 0.67; 3.5 (3–4)
**Basic Knowledge**	**Accuracy**	3.81 ± 0.39; 4 (4–4)	3.83 ± 0.30; 4 (3.63–4)	3.81 ± 0.35; 4 (3.63–4)
	**Completeness**	3.72 ± 0.43; 4 (3.5–4)	3.69 ± 0.49; 4 (3.5–4)	3.31 ± 0.55; 3.5 (3–3.5)
	**Clinical relevance**	3.81 ± 0.42; 4 (4–4)	3.83 ± 0.51; 4 (4–4)	3.86 ± 0.48; 4 (4–4)
**Diagnosis and Staging**	**Accuracy**	3.47 ± 0.69; 4 (3–4)	3.53 ± 0.72; 4 (3–4)	3.59 ± 0.64; 4 (3.38–4)
	**Completeness**	3.38 ± 0.72; 3.5 (3–4)	3.28 ± 0.60; 3.5 (3–3.63)	3.16 ± 0.72; 3.5 (2.88–3.5)
	**Clinical relevance**	3.44 ± 0.79; 4 (2.88–4)	3.59 ± 0.58; 4 (3.38–4)	3.59 ± 0.61; 4 (3–4)
**Treatment Options**	**Accuracy**	3.48 ± 0.57; 3.5 (3.13–4)	3.20 ± 0.73; 3.25 (2.63–4)	3.43 ± 0.58; 3.5 (3–4)
	**Completeness**	3.23 ± 0.59; 3 (3–3.88)	2.95 ± 0.63; 3 (2.5–3.38)	2.91 ± 0.45; 3 (2.5–3)
	**Clinical relevance**	3.18 ± 0.66; 3 (3–4)	3.09 ± 0.72; 3 (2.5–3.88)	3.20 ± 0.63; 3.25 (3–3.5)
**Prognosis**,** Risks**,** and Follow-up**	**Accuracy**	3.52 ± 0.73; 4 (3–4)	3.48 ± 0.73; 4 (3–4)	3.59 ± 0.62; 4 (3–4)
	**Completeness**	3.26 ± 0.72; 3 (3–4)	3.15 ± 0.63; 3 (3–3.5)	2.72 ± 0.58; 3 (2.5–3)
	**Clinical relevance**	3.46 ± 0.69; 4 (3–4)	3.30 ± 0.63; 3 (3–4)	3.11 ± 0.66; 3 (3–3.75)

**Table 3:** B. Model-based comparisons

Outcome	Overall platform effect	Platform × domain interaction	Significant post hoc comparisons
**Accuracy**	χ²(2) = 3.79, *p* = 0.150	χ²(6) = 5.91, *p* = 0.434	None
**Completeness**	χ²(2) = 55.36, *p* < 0.001	χ²(6) = 11.43, *p* = 0.076	ChatGPT > Claude: OR 1.98 (95% CI 1.17–3.35), *p* = 0.011; ChatGPT > Gemini: OR 7.01 (4.02–12.23), *p* < 0.001; Claude > Gemini: OR 3.54 (2.10–5.97), *p* < 0.001
**Clinical relevance**	χ²(2) = 1.13, *p* = 0.568	χ²(6) = 12.84, *p* = 0.046	Prognosis domain: ChatGPT > Gemini, OR 5.05 (95% CI 1.89–13.47), adjusted *p* = 0.015

Values are presented as mean ± standard deviation; median (interquartile range). Higher scores indicate better performance. Descriptive statistics are based on the primary response generated for each question on each platform.

Inferential analyses were performed using cumulative link mixed models based on the original reviewer-level 1–4 ordinal ratings. Platform and clinical domain were included as fixed effects, reviewer as a fixed effect, and question as a random intercept. Overall platform effects and platform-by-domain interactions were assessed using likelihood-ratio tests. Pairwise platform comparisons were adjusted for multiplicity using the Holm method. For clinical relevance, Holm correction was applied across all 12 domain-specific platform comparisons. OR, proportional odds ratio; CI, confidence interval.

### Readability (expert and automated assessment)

Expert-rated readability differed significantly across platforms in the cumulative link mixed model (likelihood-ratio χ² (2) = 54.76, *p* < 0.001). Gemini achieved the highest descriptive expert readability score (mean 2.87 ± 1.00), followed by ChatGPT (2.56 ± 1.15) and Claude (2.55 ± 1.15) (Table 4a; ). In Holm-adjusted pairwise comparisons, Gemini had higher odds of receiving a higher readability rating than ChatGPT (OR 6.69, 95% CI 3.54–12.63; *p* < 0.001) and Claude (OR 7.57, 95% CI 3.98–14.39; *p* < 0.001), whereas ChatGPT and Claude did not differ significantly (OR 1.13, 95% CI 0.63–2.03; *p* = 0.680). There was no significant platform-by-domain interaction (χ² (6) = 2.70, *p* = 0.846) (Table 5b).

Automated readability assessment using the Flesch–Kincaid Grade Level also revealed significant differences among platforms (Friedman χ² = 9.684, *p* = 0.008). Gemini responses required the lowest reading grade level (10.93 ± 1.67), followed by Claude (11.24 ± 2.25) and ChatGPT (11.83 ± 2.02). Across all models, generated content corresponded to approximately 10th–12th grade reading levels.

Expert readability ratings showed significant inverse correlations with Flesch–Kincaid Grade Levels across all platforms (ChatGPT: ρ = −0.343, *p* = 0.002; Claude: ρ = −0.330, *p* = 0.003; Gemini: ρ = −0.290, *p* = 0.010) (Table 6).

**Fig. 1:**
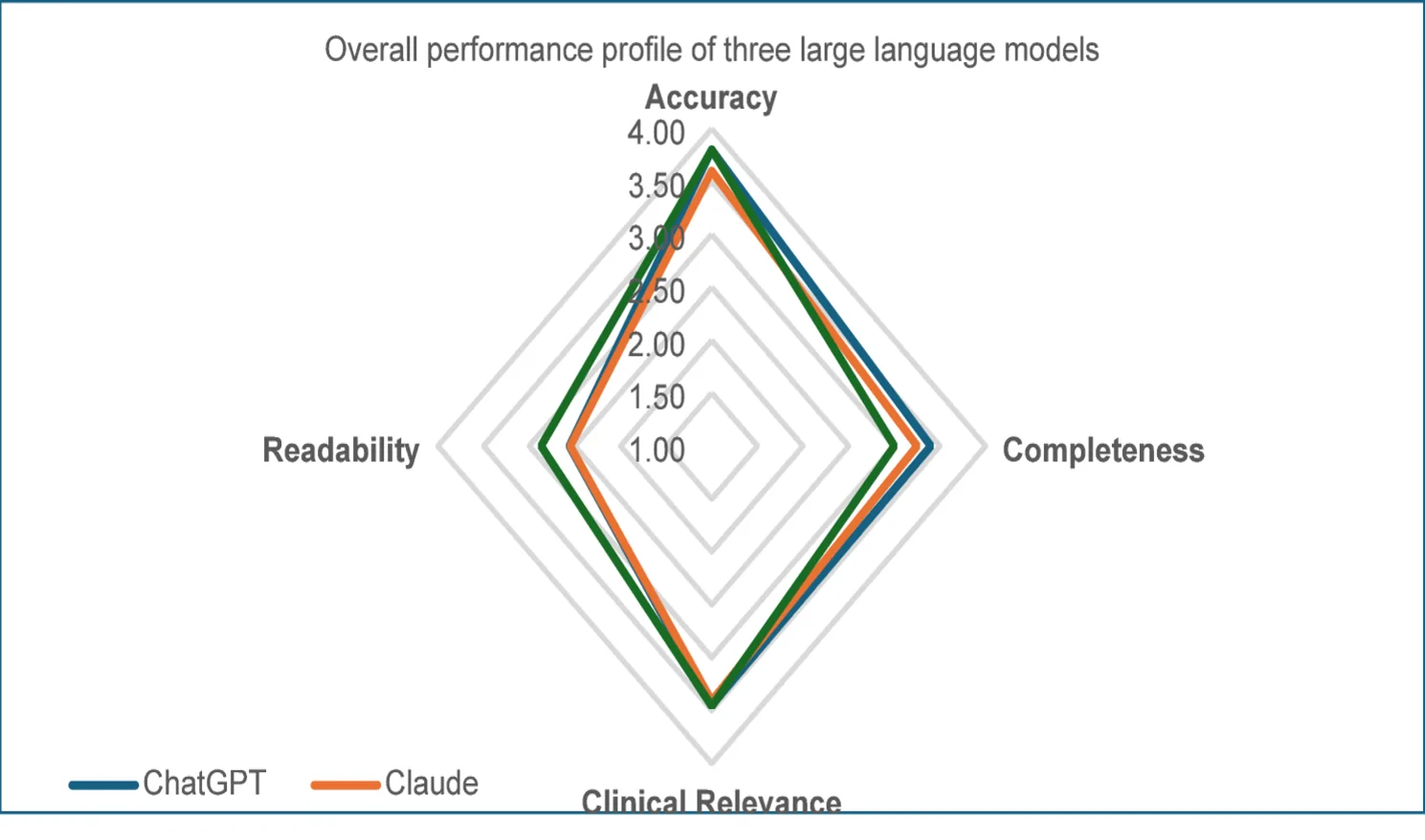
Radar visualization of accuracy, completeness, readability, and clinical relevance across LLMs

The multidimensional performance profiles of all large language models are illustrated in Fig. 1.

**Table 4:** a. Readability scores of ChatGPT, Claude and Gemini across overall and domains

Domain	ChatGPT	Claude	Gemini
**Overall**	2.56 ± 1.15; 3 (1.5–4)	2.55 ± 1.15; 3 (1–3.75)	2.87 ± 1.00; 3 (2–4)
**Basic Knowledge**	2.86 ± 0.94; 3 (2.25–3.38)	2.75 ± 0.97; 3 (2.25–3)	3.17 ± 0.77; 3 (3–4)
**Diagnosis and Staging**	2.53 ± 1.12; 2.75 (1.5–3.5)	2.59 ± 1.00; 3 (2–3.13)	2.97 ± 0.96; 3 (2.38–4)
**Treatment Options**	1.98 ± 1.03; 2 (1–2.75)	2.02 ± 0.99; 2 (1–2.88)	2.39 ± 0.87; 2 (2–3)
**Prognosis**,** Risks**,** and Follow-up**	2.91 ± 1.27; 4 (1.75–4)	2.87 ± 1.39; 4 (1–4)	3.04 ± 1.19; 4 (2–4)

**Table 5:** B. Model-Based Analysis of Expert-Rated Readability

Analysis	Result
**Overall platform effect**	χ²(2) = 54.76, *p* < 0.001
**Platform × domain interaction**	χ²(6) = 2.70, *p* = 0.846
**Gemini vs. ChatGPT**	OR 6.69 (95% CI 3.54–12.63), *p* < 0.001
**Gemini vs. Claude**	OR 7.57 (95% CI 3.98–14.39), *p* < 0.001
**ChatGPT vs. Claude**	OR 1.13 (95% CI 0.63–2.03), *p* = 0.680

Values are presented as mean ± standard deviation; median (interquartile range). Readability was rated on a four-point ordinal scale, with higher scores indicating greater readability. Descriptive statistics are based on the primary response generated for each question on each platform.

Inferential analysis was performed using a cumulative link mixed model based on the original reviewer-level 1–4 ordinal readability ratings. Platform and clinical domain were included as fixed effects, reviewer as a fixed effect, and question as a random intercept. The overall platform effect and platform-by-domain interaction were assessed using likelihood-ratio tests. Pairwise platform comparisons were adjusted for multiplicity using the Holm method. OR, proportional odds ratio; CI, confidence interval.

**Table 6:** Comparison of Expert and Automated Readability Assessments

Platform	Expert Readability (1–4 scale)	Flesch–Kincaid Grade Level	Correlation (ρ)	*p*-value
ChatGPT	2.56 ± 1.15	11.83 ± 2.02	-0.343	0.002
Claude	2.55 ± 1.15	11.24 ± 2.24	-0.330	0.003
Gemini	2.87 ± 1.00	10.92 ± 1.67	-0.290	0.010
p-value		0.008	-	-

Values are presented as mean ± standard deviation. Expert readability was rated on a four-point ordinal scale (1 = very difficult; 4 = easy). Flesch–Kincaid Grade Level represents the estimated U.S. grade level required for comprehension. The overall between-platform comparison of Flesch–Kincaid Grade Levels was performed using the Friedman test. Associations between expert readability ratings and Flesch–Kincaid Grade Levels were assessed using Spearman’s rank correlation coefficient. Negative correlations indicate that lower grade levels (easier reading) correspond to higher expert readability ratings. Model-based comparisons of expert readability are presented separately in Table 5B.

**Fig. 2:**
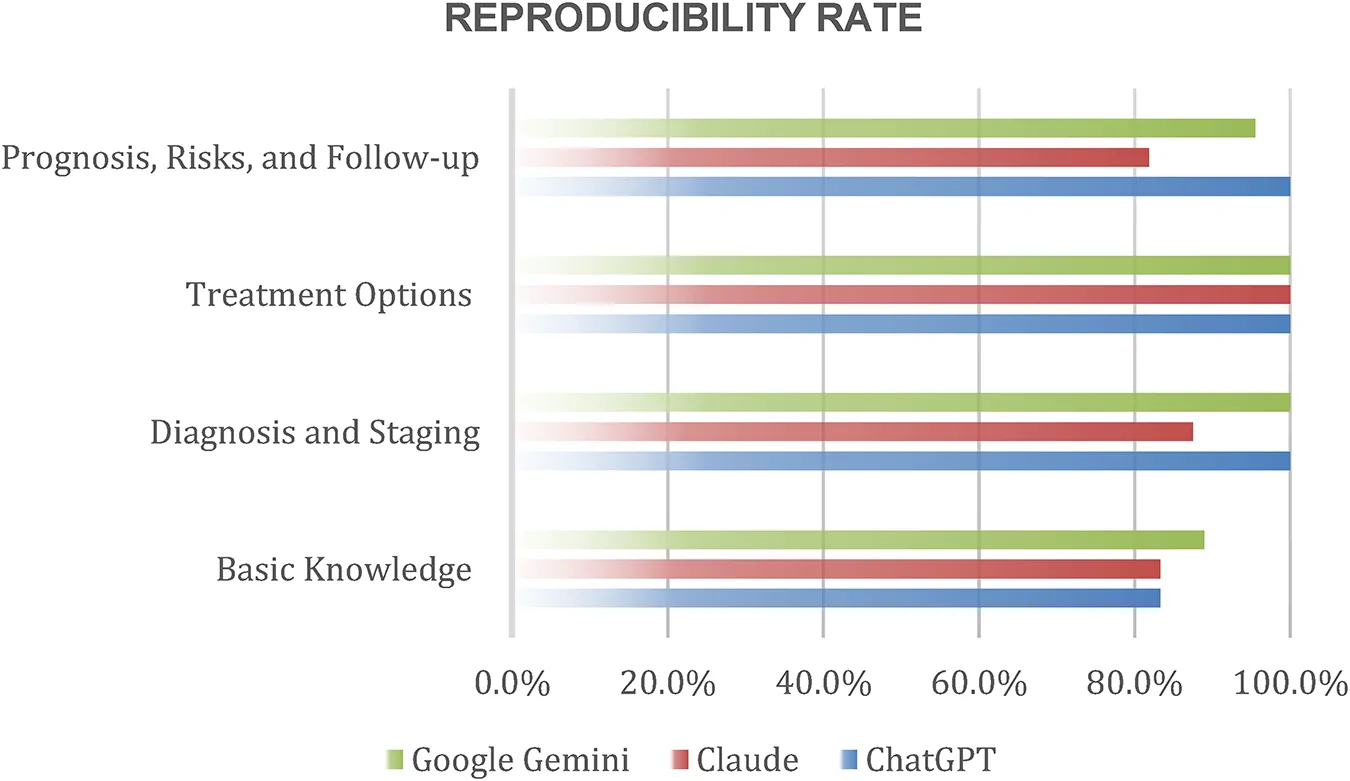
Reproducibility rates of AI-generated responses across question categories for the three platforms. Bar chart showing reproducibility rates of AI-generated responses across thematic question domains for ChatGPT, Claude, and Gemini. Reproducibility was defined as clinically equivalent responses across repeated iterations. Values represent the percentage of reproducible responses in each domain

Overall reproducibility rates were 76/79 (96.2%; 95% CI 89.4–98.7%) for ChatGPT, 70/79 (88.6%; 95% CI 79.7–93.9%) for Claude, and 76/79 (96.2%; 95% CI 89.4–98.7%) for Gemini. In the GEE logistic model accounting for clustering of platform-specific observations within questions, the overall platform effect did not reach statistical significance (Wald χ² (2) = 5.26, *p* = 0.072). Compared with ChatGPT, Claude showed numerically lower odds of reproducibility (OR 0.29, 95% CI 0.07–1.21), whereas Gemini showed no difference (OR 1.00, 95% CI 0.18–5.60). Platform-by-domain interaction estimates were not interpreted because sparse non-reproducible events and complete separation in the prognosis domain resulted in unstable estimates (Fig. 2).

**Fig. 3:**
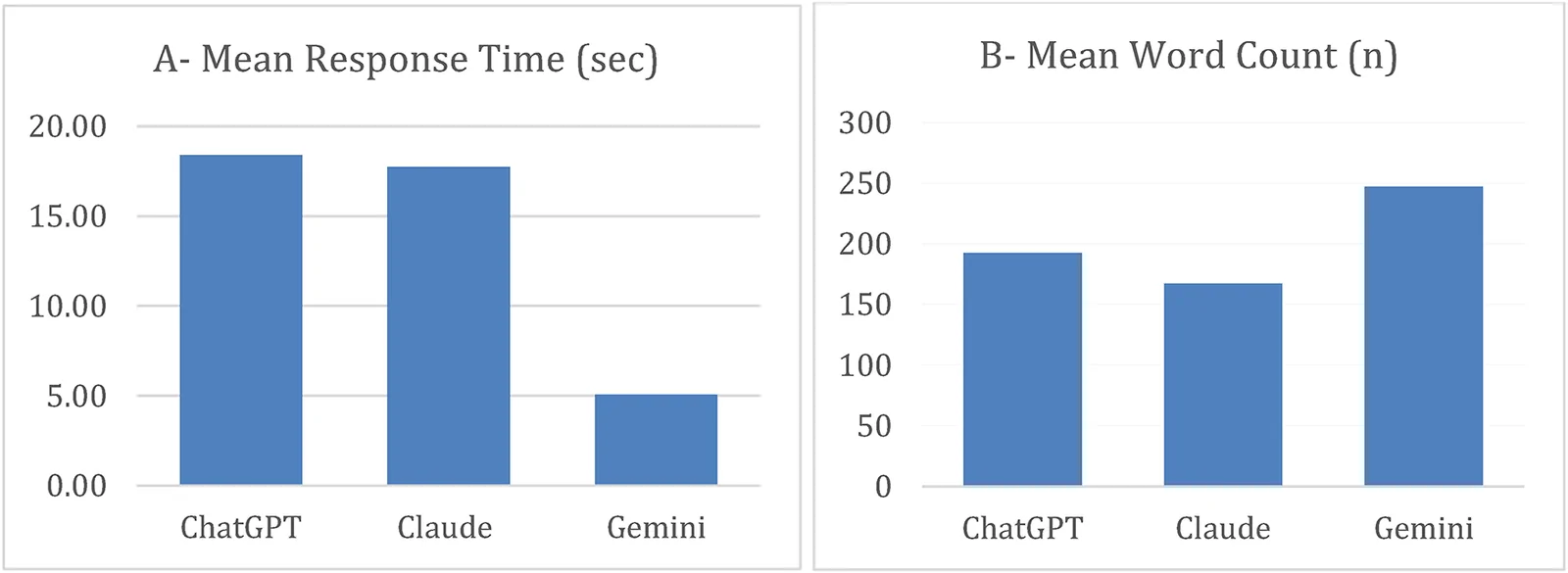
Response Efficiency Metrics Across Large Language Models. Response efficiency metrics across ChatGPT, Claude, and Gemini. (**A**) Mean response time in seconds per question. (**B**) Mean word count per response. These metrics were used to compare response speed and response length across platforms

The response time of the models differed significantly. Mean response time was 18.4 ± 9.86 s for ChatGPT (range 5.72–50.47 s), 17.74 ± 4.85 s for Claude (range 10.96–45.41 s), and 5.05 ± 1.01 s for Gemini (range 2.78–7.13 s). Friedman analysis demonstrated a statistically significant difference among the three models (χ² = 118.557, *p* < 0.001). Gemini generated responses substantially faster than both ChatGPT and Claude, whereas ChatGPT and Claude showed comparable response times (Fig. 3A).

The word count of AI-generated responses differed significantly between models. The mean word count was 192.58 ± 60.64 words for ChatGPT (range 63–503 words), 166.99 ± 55.53 words for Claude (range 95–545 words), and 247 ± 81 words for Gemini (range 41–431 words). Friedman test showed a statistically significant difference among the three models (χ² = 67.09, *p* < 0.001)(Fig. 3B).

### Correlation analysis between response characteristics and quality scores

Across all three models, response length showed a consistent inverse correlation with readability (Spearman’s ρ ranging from − 0.305 to − 0.398, all *p* < 0.01). Word count was not significantly associated with completeness or clinical relevance for any platform. No significant association between word count and accuracy was observed for ChatGPT or Gemini; however, Claude showed a modest inverse correlation between word count and accuracy (ρ = −0.323, *p* = 0.004). Strong positive correlations were observed among accuracy, completeness, and clinical relevance scores within each model (ρ = 0.42–0.76), indicating that these quality dimensions were related but not interchangeable (Table 7).

**Table 7:** Spearman’s rank correlation between response length and evaluation metrics across large language models

Model	Word Count - Accuracy (*p*)	Word Count - Completeness (*p*)	Word Count - Clinical Relevance (*p*)	Word Count - Readability (*p*)
**ChatGPT**	−0.117 (*p* = 0.304)	−0.170 (*p* = 0.134)	−0.171 (*p* = 0.131)	−0.359 (*p* = 0.001)
**Claude**	−0.323 (*p* = 0.004)	−0.144 (*p* = 0.206)	−0.100 (*p* = 0.378)	−0.398 (*p* < 0.001)
**Gemini**	−0.048 (*p* = 0.672)	0.063 (*p* = 0.582)	−0.123(*p* = 0.278)	−0.305 (*p* = 0.006)

Values are Spearman’s rank correlation coefficients (ρ), with corresponding two-sided *p*-values in parentheses. Correlation analyses were considered exploratory and were not adjusted for multiple comparisons.

## Discussion

In this multi-platform evaluation of AI-generated responses to patient-oriented lung cancer questions, all three models demonstrated high accuracy and clinical relevance. Mixed-effects analyses showed no significant overall platform difference in accuracy, whereas completeness and expert-rated readability differed significantly across platforms. Clinical relevance showed no overall platform effect but demonstrated a significant platform-by-domain interaction, indicating that relative performance varied according to the clinical context. Reproducibility was high across all platforms, with Claude showing numerically lower consistency than ChatGPT and Gemini, although the overall platform effect did not reach statistical significance. All models generated content at 10th–12th grade reading levels, exceeding the recommended 6th–8th grade standard for patient education. Together, these findings demonstrate that similar overall accuracy does not imply equivalent performance across other clinically relevant dimensions of patient-oriented AI communication.

Our findings should be interpreted within a rapidly expanding comparative literature. Recent studies have moved beyond single-model evaluations and increasingly compared multiple contemporary LLMs across patient-oriented outcomes, including accuracy, readability, completeness, understandability, actionability, and information quality. In lung cancer, Nguyen et al. compared five publicly available LLMs using standardized questions and demonstrated model-dependent differences in response accuracy, while Önal and Temiz Bekce compared ChatGPT, Gemini, and Copilot across readability, quality, understandability, and actionability. Similar multi-model evaluations across other cancer settings have likewise demonstrated that performance may vary substantially across quality dimensions. Our findings extend this literature by applying a uniform question set across three contemporary LLMs and jointly evaluating accuracy, completeness, clinical relevance, readability, and repeated-response consistency [6–9, 15, 16]. 

An important finding was the dissociation between completeness and clinical relevance. Completeness differed significantly across platforms, with ChatGPT showing higher odds of receiving a higher completeness rating than Claude and Gemini, and Claude showing higher odds than Gemini. In contrast, there was no overall platform effect on clinical relevance. However, the significant platform-by-domain interaction indicates that clinical relevance was not entirely uniform across clinical contexts; after adjustment for multiple comparisons, ChatGPT received higher clinical relevance ratings than Gemini within the prognosis domain, whereas no other domain-specific comparison remained significant. Notably, although Gemini generated the longest responses, greater response length did not translate into higher completeness or clinical relevance. Word count was not associated with either dimension across platforms. An exploratory inverse association between word count and accuracy was observed for Claude, while longer responses were consistently associated with lower readability ratings across all three platforms. These findings suggest that greater response length alone does not ensure more complete or clinically useful information and may instead increase linguistic complexity. Accordingly, completeness and clinical relevance should be considered as distinct dimensions when evaluating patient-oriented AI responses.

Readability was one of the dimensions showing a clear between-platform difference. Mixed-effects analysis showed significantly higher expert-rated readability for Gemini than for ChatGPT and Claude, with no significant platform-by-domain interaction. Automated assessment similarly showed lower Flesch–Kincaid Grade Levels for Gemini, and expert and automated measures were inversely correlated. Nevertheless, all models generated content at approximately 10th–12th grade reading levels, exceeding commonly recommended levels for patient education, consistent with previous studies [6, 8, 16, 17]. These findings indicate that AI-generated lung cancer information remains linguistically complex despite model-dependent differences in readability. Importantly, readability reflects linguistic complexity and should not be interpreted as direct evidence of patient comprehension, understandability, or actionability, which were not assessed in this study. Future evaluations should therefore complement readability metrics with direct patient-centered measures of understanding and usability.

Reproducibility is increasingly recognized as a distinct dimension of LLM performance in healthcare, although approaches to its assessment remain heterogeneous. Recent studies have examined response stability using repeated generations, agreement-based assessments, and semantic or statistical measures, emphasizing that factual accuracy and response consistency represent related but distinct properties of model performance. In our study, overall response consistency was high across all three platforms, reaching 96.2% for ChatGPT and Gemini and 88.6% for Claude. Although Claude showed numerically lower reproducibility, the GEE analysis accounting for repeated platform-level observations within questions did not demonstrate a statistically significant overall platform effect. Accordingly, this numerical difference should not be interpreted as evidence of inferior reproducibility for Claude. Nevertheless, clinically meaningful variation across repeated responses remains relevant because patients may re-query AI tools to verify or clarify information, and inconsistent responses to identical questions may increase uncertainty. Our study contributes to this emerging evidence by evaluating clinical equivalence across five independent responses to each of 79 patient-oriented lung cancer questions on each platform [18, 19].

From a clinical perspective, our findings suggest that contemporary LLMs may serve as supportive tools for patient education in lung cancer care, provided their limitations are acknowledged. High accuracy and clinical relevance indicate potential utility for addressing common patient questions and reinforcing information discussed during clinical encounters. However, the observed differences in completeness and readability, together with numerical variation in reproducibility, indicate that performance is not uniform across quality dimensions. These systems should therefore be positioned as clinician-supervised adjuncts rather than autonomous patient-facing tools, with appropriate oversight and readability calibration [15, 20]. Until such safeguards are established, unsupervised use may increase the risk of miscommunication in clinical contexts.

Several limitations should be acknowledged. First, evaluation was restricted to English-language questions derived from online searches and patient-oriented resources rather than directly from patients or caregivers; therefore, the findings do not directly measure patient comprehension or educational effectiveness. Second, the rapidly evolving nature of proprietary LLMs limits temporal reproducibility, as model updates and provider-side changes may alter responses over time. In addition, generation parameters such as temperature, top-p, and random seed were not user-accessible and could not be standardized. Third, assessment by two thoracic surgeons may not fully represent other clinical perspectives, and no single predefined reference standard was applicable across all questions. In addition, accuracy, completeness, clinical relevance, and readability were evaluated using a single prespecified primary response per question and platform; therefore, these assessments may not fully capture within-model variability in these quality dimensions across repeated generations. Fourth, reproducibility was assessed using a binary clinical-equivalence framework and jointly by the reviewers; therefore, it reflects model response consistency rather than the reliability of reviewer assessment and precludes estimation of independent inter-rater agreement for this outcome. Sparse non-reproducible events also limited domain-specific analyses. Finally, quantitative NLP-based similarity measures, multimodal outputs, and real-world patient outcomes were not evaluated. Readability assessment reflected linguistic complexity rather than patient comprehension, and validated measures of understandability and actionability were not included. Future studies should incorporate directly patient-derived questions and prospective patient-centered assessments of comprehension, understandability, actionability, and usefulness.

Future research should prioritize several directions. First, prospective patient-based studies are needed to complement expert clinical evaluation by assessing patient-centered outcomes such as comprehensibility, perceived usefulness, relevance to individual information needs, and confidence in AI-generated information across diverse health literacy levels. These patient-reported assessments should be complemented by objective comprehension testing using validated approaches, such as teach-back methods, to determine whether clinically accurate information is also correctly understood by patients. Second, further multilingual evaluation across diverse patient populations is warranted, as model performance may vary substantially across languages and healthcare contexts. Third, controlled experiments systematically varying prompt strategies (e.g., explicit reading level instructions) could identify optimal configurations for balancing accuracy and readability. Finally, longitudinal tracking of model performance across version updates would enable early detection of quality degradation—analogous to post-market surveillance for medical devices.

In conclusion, contemporary LLMs demonstrated high overall accuracy and clinical relevance when answering patient-oriented lung cancer questions, although differences were observed in completeness and readability. Reproducibility was high across all platforms, with no significant between-platform difference. However, all models generated content exceeding recommended patient reading levels. These findings highlight the need to consider readability, completeness, and response consistency alongside factual accuracy when evaluating patient-oriented AI information. Further patient-based validation is needed before routine clinical integration.

## Declarations

### Consent for publication

Not applicable.

This manuscript does not contain data from any individual person in any form (including individual details, images, or videos).

### Data availability

All data generated or analyzed during this study are included in this published article and its supplementary information files. The full list of questions and selected AI-generated responses, including low-accuracy and non-reproducible outputs, are provided in the Supplementary Material. Additional data are available from the corresponding author upon reasonable request.

### Competing interests

EO and GGP declare that they have no competing interests.

### Funding

This research received no external funding.

### Authors’ contributions

EO and GGP conceived and designed the study. EO performed data collection and AI querying. EO and GGP independently evaluated AI-generated responses. EO performed statistical analyses. EO drafted the manuscript. GGP critically revised the manuscript for important intellectual content. All authors read and approved the final manuscript.

### Acknowledgements

Not applicable.

### Authors’ information (optional)

EO is a thoracic surgeon with clinical and academic expertise in lung cancer surgery and patient-centered clinical research.

GGP is a thoracic surgeon with clinical experience in lung cancer management and interest in medical education and artificial intelligence–based evaluation of patient information tools.

Pairwise comparisons were derived from cumulative link mixed models. Odds ratios (ORs) greater than 1 indicate higher odds of receiving a higher ordinal rating for the first-listed platform relative to the second-listed platform. Multiplicity was controlled using the Holm method. For the significant platform-by-domain interaction in clinical relevance, Holm correction was applied across all 12 domain-specific platform comparisons. OR, proportional odds ratio; CI, confidence interval.

## Supplementary information

Below is the link to the electronic supplementary material.

Supplementary Material 1

Supplementary Material 2

Supplementary Material 3

Supplementary Material 4

## Data Availability

All data generated or analyzed during this study are included in this published article and its supplementary information files. The full list of questions and selected AI-generated responses, including low-accuracy and non-reproducible outputs, are provided in the Supplementary Material. Additional data are available from the corresponding author upon reasonable request.

## Declarations

### Background

Large language models (LLMs) are increasingly used by patients seeking lung cancer–related medical information. Recent studies have increasingly compared multiple LLMs for patient-facing medical information; however, differences in clinically relevant performance dimensions and consistency across repeated responses remain important areas for evaluation.

### Methods

Seventy-nine lung cancer–related patient questions were queried across three freely accessible LLM platforms (ChatGPT, Claude, and Google Gemini) between December 2025 and January 2026. Responses were independently evaluated by two specialist thoracic surgeons for accuracy, completeness, clinical relevance, and readability, with disagreements resolved by consensus. Reproducibility was assessed through five repeated independent queries using a clinical-equivalence framework, and automated readability was assessed using the Flesch–Kincaid Grade Level. Ordinal outcomes were analyzed using cumulative link mixed models accounting for repeated evaluation of questions, while reproducibility was analyzed using generalized estimating equations.

### Results

All platforms demonstrated high accuracy, with no significant overall platform difference (*p* = 0.150). Completeness differed significantly across platforms (*p* < 0.001), with higher ratings for ChatGPT than Claude and Gemini and for Claude than Gemini. Clinical relevance showed no overall platform effect (*p* = 0.568), although a platform-by-domain interaction was observed (*p* = 0.046). Expert-rated readability differed significantly (*p* < 0.001), with Gemini receiving higher ratings than ChatGPT and Claude. All platforms produced content requiring approximately 10th–12th grade reading levels. Reproducibility was high overall (88.6–96.2%), with no statistically significant overall platform effect (*p* = 0.072).

### Conclusions

Contemporary LLMs demonstrated high accuracy and clinical relevance, but differed in completeness and readability. Reproducibility was high across platforms, while all models exceeded recommended patient reading levels. Further patient-based validation is needed before routine clinical integration.

## Nomenclature

Fr: Darcy Forchheimer factor
$${\rm{T}}$$: Temperature
$${T}_{\infty }$$: Ambient thermal
${\rm{K}}\,$: Oxytactic microbe’s Density
Nu: Nusselt Number
$${D}_{B}$$: Mass diffusivity
$\left({P},Q\right)$: Flow fields of fluid$(m.{s}^{-1})$
Ec: Eckert number
Sb: Bio-convection Schmidt number
$${C}_{b}$$: Forchheimer coefficient
$${\upsilon }_{f}\,$$: Kinematic viscosity
$${\mu }_{f}$$: Dynamic viscosity$$({kg}{m}^{-1}{s}^{-1})$$
Ω: Microbe solutal
${K}_{\infty }$: Ambient Oxytactic microbe
$${C}_{\infty }$$: Ambient concentration
${\rm{N}}$: Microbial Density of gyrotactic
Sh: Sherwood Number
Pe: Pecklet number
Se: Oxytactic microbe Schmidt number
$${N}_{\infty }$$: gyrotactic microorganisms
Φ: Volume fraction of NPs
Kp: Darcy parameter
$${k}^{* }$$: Porosity coefficient
$${\rho }_{f}$$: Fluid density $${\boldsymbol{(}}{\rm{kg}}/{{\rm{m}}}^{3})$$
$${D}_{S}$$: Oxygen diffusivity
Sc: Schmidt number
C: Concentration for nanoparticle
Cf: Skin friction
${D}_{m}$: Gyrotactic microorganism diffusivity
$${\kappa }_{f}$$: Temperature conductivity$$(W{m}^{-1}{k}^{-1})$$
$$b{W}_{C}$$: Constants
Pr: Prandtl number
NF-: Nanofluid
LTNE: Local thermal non-equilibrium
ML: Machine learning
SB: Stefan blowing
THNF: Trihybrid nanofluid
NPs: Nanoparticles
DFF: Darcy–Forchheimer flow
BNF: Boger nanofluid
