# Supplementary material for: Assessing the reliability of AI-generated responses to patient-oriented lung cancer questions: a multi-platform study
Source: BMC Pulm Med. 2026 Oct 23;26:522. doi: 10.1186/s12890-026-04903-x (PMC13726110; doi:10.1186/s12890-026-04903-x)
Supplement: Supplementary file 1 [file 12890_2026_4903_MOESM4_ESM.docx]

| **Supplementary Table S2:** Model-Based Post Hoc Pairwise Comparisons Across AI Platforms**Outcome / Domain** | **Comparison** | **OR** | **95% CI** | **Holm-adjusted *p*** |
| --- | --- | --- | --- | --- |
| **Completeness – Overall** | **ChatGPT vs Claude** | 1.98 | 1.17–3.35 | 0.011 |
|  | **ChatGPT vs Gemini** | 7.01 | 4.02–12.23 | <0.001 |
|  | **Claude vs Gemini** | 3.54 | 2.10–5.97 | <0.001 |
| **Clinical relevance – Prognosis, Risks, and Follow-up** | **ChatGPT vs Claude** | — | — | — |
|  | **ChatGPT vs Gemini** | 5.05 | 1.89–13.47 | 0.015 |
|  | **Claude vs Gemini** | — | — | — |
| **Readability – Overall** | **Gemini vs ChatGPT** | 6.69 | 3.54–12.63 | <0.001 |
|  | **Gemini vs Claude** | 7.57 | 3.98–14.39 | <0.001 |
|  | **ChatGPT vs Claude** | 1.13 | 0.63–2.03 | 0.680 |

Pairwise comparisons were derived from cumulative link mixed models. Odds ratios (ORs) greater than 1 indicate higher odds of receiving a higher ordinal rating for the first-listed platform relative to the second-listed platform. Multiplicity was controlled using the Holm method. For the significant platform-by-domain interaction in clinical relevance, Holm correction was applied across all 12 domain-specific platform comparisons. OR, proportional odds ratio; CI, confidence interval.
